# Chemically Recyclable and Biodegradable Vulcanized Rubber

**DOI:** 10.1021/acssuschemeng.3c08435

**Published:** 2024-04-11

**Authors:** Simon T. Schwab, Taylor F. Nelson, Stefan Mecking

**Affiliations:** Chair of Chemical Materials Science, Department of Chemistry, University of Konstanz, Universitätsstraße 10, 78464 Konstanz, Germany

**Keywords:** vulcanizates, recycling, biodegradation, elastomers, sustainable materials

## Abstract

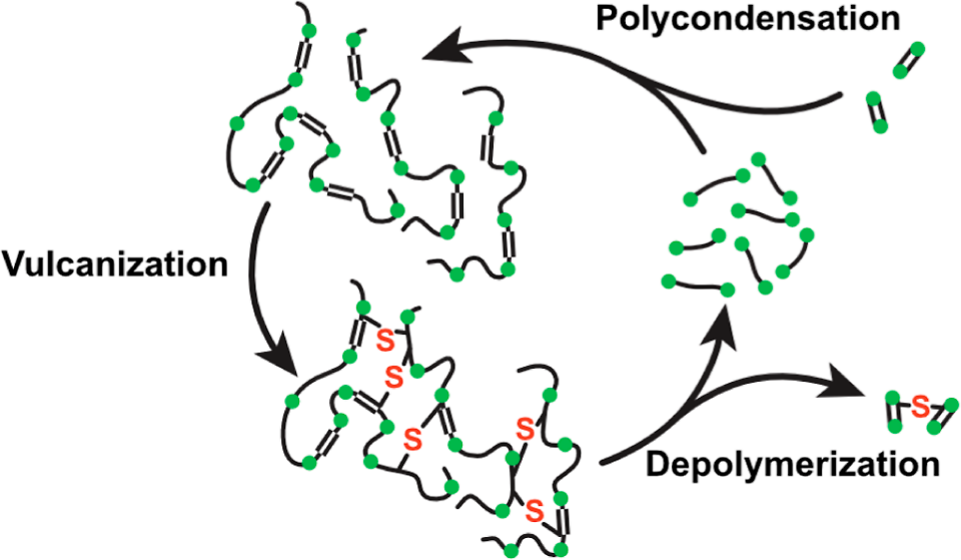

The cross-linked nature of vulcanized rubbers as used in tire and many other applications prohibits an effective closed-loop mechanical or chemical recycling. Moreover, vulcanization significantly retards the material’s biodegradation. Here, we report a recyclable and biodegradable rubber that is generated by the vulcanization of amorphous, unsaturated polyesters. The elastic material can be broken down *via* solvolysis into the underlying monomers. After removal of the vulcanized repeat units, the saturated monomers, constituting the major share of the material, can be recovered in overall recycling rates exceeding 90%. Respirometric biodegradation experiments by ^13^CO_2_ tracking under environmental conditions *via* the polyesters’ diol monomer indicated depolymerization and partial mineralization of the vulcanized polyester rubbers.

## Abstract 2

Polyesters from saturated and unsaturated monomers can be vulcanized to rubbers and chemically recycled *via* solvolysis to their monomers.

## Introduction

Rubbers, or vulcanizates, are among the polymeric materials with the largest production volume, with an annual output of both natural and synthetic rubber exceeding 29 million metric tons.^1^ Their performance and ease of production render them well-suited for application in, *e.g.*, tires, seals, and gloves.^2−4^ The excellent elastomeric properties and durability of rubbers arise from the vulcanization process, which creates a covalent network between the polymer chains through sulfur bonds. However, this stability, while advantageous for material properties, hampers recycling and decelerates their biodegradation.^5,6^

Due to their cross-linked nature, rubbers are intractable and insoluble, rendering them inherently unamenable to recycling through conventional methods involving material flow.^6^ Consequently, a large share of the one billion waste tires exceeding their operational lifespan every year, representing the biggest source of waste rubber, are landfilled or burned.^7,8^ Current reuse practices mainly involve the grinding of waste rubber to particles for subsequent use primarily as fillers in asphalt and in cement production.^9,10^ To a limited extent, these shredded rubber particles can be added to virgin rubber in the production of new tires or other products.

Chemical recycling of vulcanizates encompasses processes such as pyrolysis, yielding so-called “tire-derived fuel”,^11^ as well as devulcanization,^12−14^ which allows the further usage of the rubber as well as potential fillers or reinforcing materials. Devulcanization involves the selective breaking of the sulfur cross-links by chemical, biological, or physical means, such as thermal or mechanical treatment. However, the parallel breaking of rubber polymer chains under the harsh conditions required unavoidably compromises the material properties of the resulting devulcanized rubbers. Therefore, only a minor share of devulcanized rubber can be added to the virgin material without compromising the overall material properties.

Vulcanizates may end up in the environment by different pathways, *e.g.*, mismanagement of waste or shedding of tire wear particles. While natural rubber is readily biodegradable,^5^ vulcanization significantly retards this process by adding bonds that need to be cleaved to yield bioavailable low-molecular-weight products. Furthermore, the cross-linked nature of vulcanized rubber decreases the accessibility of enzymes to cleavable double bonds in the polymer chains.^15^

A variety of concepts exist for creating recyclable and/or degradable elastomers, the most prominent example being thermoplastic elastomers, based on the formation of reversible noncovalent cross-links. These materials combine remarkable mechanical properties with (re)processability and the potential of using renewable feedstocks.^16^ Nonetheless, the lack of covalent cross-links leads among others to lower thermal stability and abrasion resistance, as well as stress softening, limiting their utility for certain applications.^17^ The creation of reversible covalently cross-linked networks is another widely studied approach,^18−20^ with vitrimers^21^ being a prominent example. However, the synthesis of these materials necessitates intricate, multistep procedures, leading so far to costly materials. Instead of reversible cross-links, the reversible bonds enabling recycling or biodegradation can also be located in the polymer backbone itself. Networks of amorphous polyesters synthesized with multifunctional monomers exhibit rubber-like properties and are chemically recyclable, as demonstrated elegantly by the Hillmyer group.^22,23^ Yet, their processing markedly differs from vulcanization of rubbers.

The vulcanization of unsaturated, amorphous polyesters would ideally combine the processing and material properties of rubbers with the recyclability and degradability of polyesters.^24,25^ The general feasibility of vulcanizing biobased polyesters was demonstrated among others by Cramail *et al.*,^26^ Fuller *et al.*,^27^ and Matsumura *et al.*(28) with unsaturated polyhydroxyalkanoates and ricinoleic acid-based polyesters. The recyclability of these materials or their biodegradation was not addressed, however.

We now report amorphous polyesters generated by A_2_ + B_2_ polycondensation of commercially available saturated and unsaturated comonomers and their sulfur vulcanization to elastomeric rubbers and demonstrate their deconstruction *via* solvolysis for chemical recycling to the monomer, (abiotic) hydrolysis, and environmental biodegradation with isotope-selective CO_2_ tracking.

## Experimental Section

### Materials

Ethylene glycol (EG, ≥99.5%) and disodium hydrogen phosphate dihydrate (≥98%) were purchased from Carl Roth, hexenedioic acid (HA, >98%) from TCI, maleic acid (MA, 99%) and sodium dihydrogen phosphate monohydrate (≥99.99%) from Merck, *N*-tertbutyl benzothiazole sulfonamide (TBBS) and 2,2-dibutyl diethylmalonate (98%) from abcr, methanol (≥99.8%), dibutyl tin oxide (DBTO), 2,6-di-*tert*-butyl-*p*-cresol (BHT, 99%), and ^13^C_2_–EG (99 atom % ^13^C) from Sigma-Aldrich, and chloroform (≥99.8%) from VWR. Deuterated solvents were obtained from Eurisotop and dried over molecular sieves from Riedel-de Haën (0.4 nm). Pripol 1009 (dimer acid, DA, 98%) was kindly provided by Croda/Cargill.

### Characterization Techniques

Differential scanning calorimetry (DSC) was performed on a Netzsch DSC 204 F1 instrument with the software Netzsch Proteus Thermal Analysis, version 6.1.0. Data reported for melting points (*T*_m_) are from the second heating cycle with a heating/cooling rate of 10 K min^–1^ and for glass transition temperatures (*T*_g_) from the maximum of the derivative of the second heating cycle using a heating/cooling rate of 30 K min^–1^.

Nuclear magnetic resonance (NMR) spectroscopy was performed on a Bruker Avance III HD 400 or Bruker Avance III 400 spectrometer. Chemical shifts were referenced to the solvent signals. Mestrenova software Mestrelab Research S.L. (version 14.1.2) was used for data evaluation.

Molecular masses of polymers were determined *via* size-exclusion chromatography (SEC) in chloroform at 35 °C on a PSS SECcurity^2^ instrument, equipped with PSS SDV linear M columns (2 × 30 cm, additional guard column) and a refractive index detector (PSS SECcurity^2^ RI). A flow rate of 1 mL/min was used. Data was evaluated *versus* polystyrene standards using the software PSS WinGPC, version 8.32.

Tensile tests were performed on a Zwick Z005/1446 Retroline tC II instrument at a crosshead speed of 5 mm min^–1^ on a tensile testing specimen (ISO 527-2, type 5A) obtained by vulcanization in a mold (*vide infra*). The tensile testing samples were preconditioned to ambient conditions for a minimum of 1 day prior to the measurement. Young’s modulus was determined at a crosshead speed of 0.5 mm min^–1^. Cyclic hysteresis tests were performed on individual test specimens for 10 consecutive cycles of loading and unloading to a constant strain of 20 and 50% with a crosshead speed of 5 mm min^–1^. The software testXpert from Zwick Roell, version 11.0, was used for data evaluation.

### Polymerization Experiments

An amorphous polyester consisting only of DA and EG was synthesized according to an established melt polycondensation protocol.^29^ This protocol was adapted for the synthesis of amorphous and unsaturated polyesters. Exemplarily, the synthesis of the polyester **H1** from DA, EG, and HA is described. DA (5.70 g, 10 mmol, 1 equiv), HA (1.44 g, 10 mmol, 1 equiv), EG (2.48 g, 40 mmol, 4 equiv), BHT (96 mg, 1 wt %), and DBTO (50 mg, 0.20 mmol, 2 mol %) were added into a round-bottom flask. The mixture was thoroughly degassed, stirred with a PTFE-coated stirring bar, and heated to 140 °C. Evolving water was distilled off. After 4 h, vacuum was gradually applied to facilitate the removal of condensate and excess EG, reaching 0.05 mbar after 24 h, while the viscosity of the reaction mixture increased. After 24 h at 0.05 mbar and 140 °C, the temperature was increased to 160 °C for 2 h and the reaction subsequently stopped by letting the reaction mixture cool down to room temperature. The uncured amorphous and unsaturated polyester was obtained as yellowish, highly viscous liquid without further workup.

Additional polyesters were prepared using MA as the unsaturated monomer. Various ratios of unsaturated to saturated monomers were utilized to produce polyesters with varying double-bond densities. The synthesis was carried out in a reactor equipped with individual glass inlets and stirring bars, enabling the simultaneous small-scale synthesis (1–2 g) of up to eight polymers. All polymers were fully amorphous. The molecular weight determined *via* NMR and SEC as well as the exact monomer composition, unreacted double bonds, and theoretical C atom recycling rates are shown in Table S1. The theoretical recycling rate was calculated with the following equation
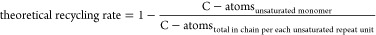
1

### Vulcanization Experiments

For vulcanization, the uncured, unsaturated polyesters were mixed in PTFE vials at 100–120 °C with 2.75 phr sulfur, 0.8 phr TBBS, and 5 phr ZnO, where phr denotes parts per 100 rubber. The homogeneous white compounds were manually inserted into a PTFE mold and heated for 1 h to 180 °C. Vulcanized materials are indicated with V, *e.g.*, **H1-V**. These elastomers were obtained as nonsticky, yellow-brownish tensile testing specimens with a faint sulfur odor, akin to vulcanized natural or synthetic rubber (Figure S1).

For determining the vulcanization time of different rubbers, a small amount of uncured polyester (50–100 mg) was added to a glass vial together with the curing agents as outlined above. The materials were mixed at 100 °C and subsequently vulcanized at 180 °C. The vulcanization time was defined as the point in time at which the sample visibly transitioned from a liquid to an elastic state.

### Gel Content Determination

Triplicate extractions of the sol fractions were conducted in CHCl_3_ to assess the gel content of the vulcanized rubber **H1-V** sample. Approximately 40 mg of the sample was immersed in 8 mL of CHCl_3_ for 6 h and shaken at 37 °C. The solution was filtered, the solvent was removed in vacuum, and the remaining mass was measured. The sol fraction was calculated from the ratio of the remaining and the initial mass. The insolubility of the vulcanized rubber even in 100 °C toluene is shown in Figure S3.

### Recycling Experiments

Methanolysis of the vulcanized rubber **H1-V** tensile testing specimen was carried out without any pretreatment of the elastomeric material. The rubber (23 g) was immersed in 300 mL of MeOH and stirred at 150 °C for 6 days in a pressure reactor without the addition of a catalyst. The resultant brown solution (Figure S4, left) was filtered to remove solids such as ZnO particles. The solvent was removed from the filtrate under vacuum, and the monomer solution was distilled. The first fraction was collected at a pressure of 5 mbar at 110–130 °C and the second fraction at 0.05 mbar and 220 °C. The last remaining monomers were distilled *via* short path distillation at 0.05 mbar and 250–300 °C. The first fraction was an EG-rich mixture (Figure S7), the second fraction mainly consisted of the cross-linked or uncross-linked HA, and in the last fraction, >99% pure (determined from NMR, Figures S5 and S6) dimer dimethyl ester was isolated in 97% yield (14.6 g, Figure S4, right).

### Hydrolytic Stability

The hydrolytic stability of polyester consisting of DA, EG, and HA was tested in phosphate buffer solution (pH = 7.2, 50 mM). Around 15 mg of uncured polyester **H1** was immersed in 10 mL of phosphate buffer solution, heated up to 75 °C, and stirred for 1 day. Subsequently, the polyester was removed from the buffer, dried in vacuum, and analyzed *via* SEC. A control sample was processed the same way but aged at 25 °C.

### Biodegradation

Polycondensation and vulcanization of a rubber equivalent in composition to **H1-V** containing DA, HA, and ^13^C labeled EG as well as vulcanization agents were carried out as described above. ^13^C-labeled samples are indicated below by *. After the vulcanization, the final material consisted of 66.95 wt % carbon based on calculations of the components used in the preparation. Of all carbon atoms present in the material, 8.61% theoretically belonged to the utilized EG, corresponding to an estimated calculated ^13^C-content of 9.62 atom %. The vulcanized rubber was manually cut into small particles (approximately 1 × 1 × 1 mm = 1 mm^3^) for addition to the soil. Additionally, ^13^C-enriched cellulose [prepared as a mixture of fully ^13^C-labeled cellulose (IsoLife, Netherlands) and unlabeled cellulose (Merck, Germany) to yield a cellulose with overall ^13^C-content of approximately 5 atom %] was prepared as a reference biodegradable polymer.

Soil mineralization experiments were conducted using a flow-through incubation system coupled to an isotope-selective cavity ring-down spectroscopy analyzer (model G2201-*i*; Picarro, USA) that has been previously described in detail.^30^ The biodegradation of each material was conducted in triplicate incubation bottles and compared to triplicate control incubation bottles to which no material was added, all held at 25 °C in the dark. The data on biodegradation of cellulose as a reference biodegradable polymer in the same soil has been published previously.^31^ The soil used (standard soil type 6S from LUFA Speyer, Germany) was characterized as a clayey loam (DIN classification based on particle size) and contained 1.5 wt % organic C, 0.17 wt % N, and had a pH of 7.3. Upon collection, the soil was sieved to 2 mm and stored at 4 °C between receiving and preparing it for incubations. Before the addition of **H1-V** or cellulose, the soils were brought to 25 °C, and the water content in each bottle was adjusted to 50% of the maximum soil water holding capacity (WHC_max_ = 40.1 g H_2_O g^–1^ dry soil), and the soils were preincubated for 1 week in order for the background soil respiration rate to equilibrate to the new conditions. Throughout the incubation, the bottles were intermittently removed from the flow-through incubation system and stored open in boxes with humidified air. The water contents of the soils were periodically readjusted gravimetrically to account for evaporative losses. Concentrations of ^12^CO_2_ and ^13^CO_2_ in the efflux gas of the bottles were measured for 15 min at a time, with automatic switching between bottles, and the data from the last 5 min of each interval averaged to serve as single time points. This sample sequence was operated continuously, with periodic measuring of synthetic air with known concentrations and isotopic signatures of CO_2_ to serve as drift correction during continuous instrument operation. Using the measured concentrations of ^12^CO_2_ and ^13^CO_2_ along with the total carbon and ^13^C contents of the added polymers, polymer–^13^C mineralization rates and subsequently, extents, were calculated according to a previously published protocol.^30^

## Results and Discussion

### Material and Recycling

**Figure 1:**
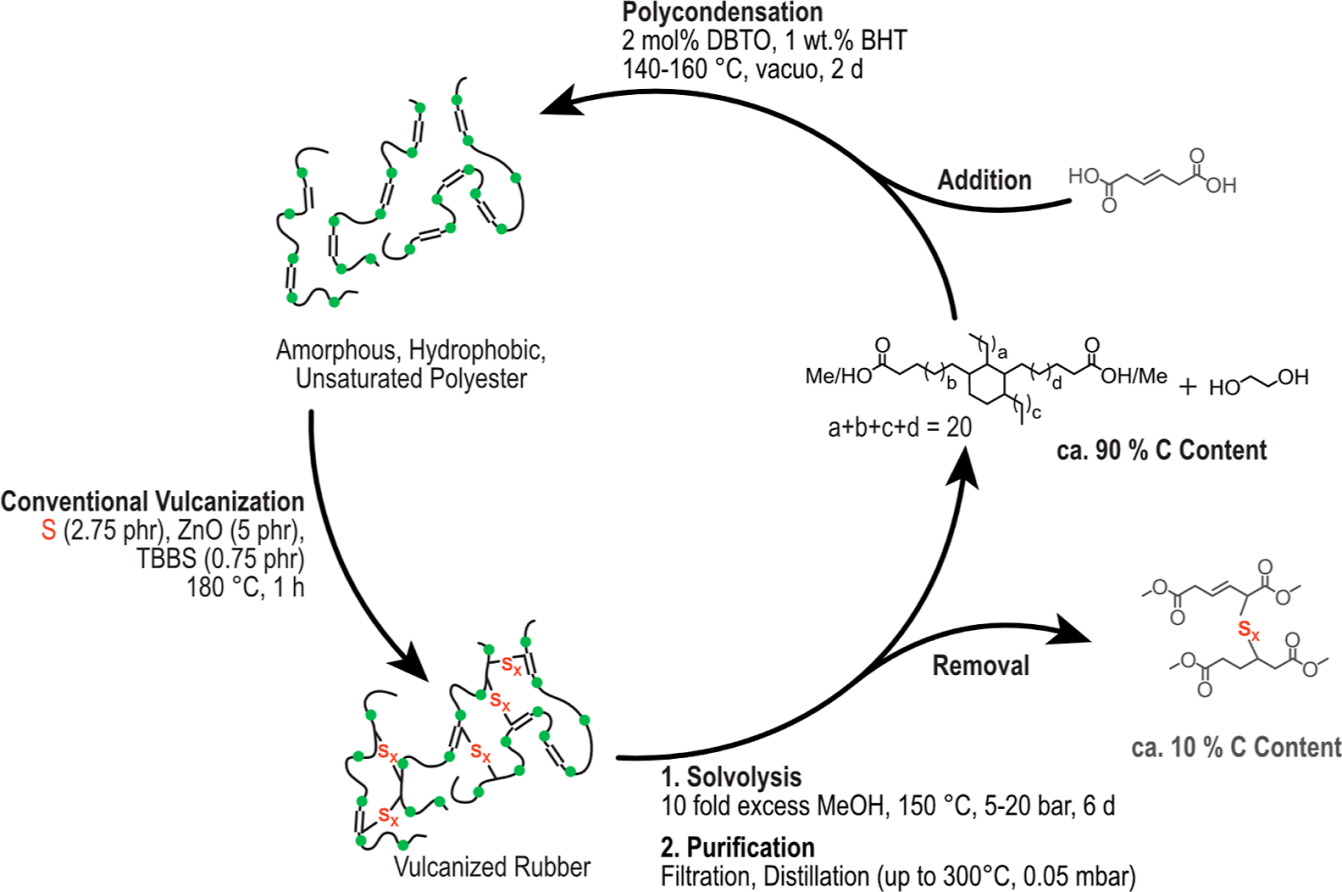
Polycondensation of saturated (DA and EG) and unsaturated (in this case: HA) monomers to amorphous, unsaturated polyesters (top), their vulcanization to rubbers (left), and their solvolysis and purification (bottom) back to the monomers. The green dots represent ester groups as predetermined breaking points in the chain. The unsaturated monomers usually comprise around 10% of the carbon atoms in the rubber polymer.

**Figure 2:**
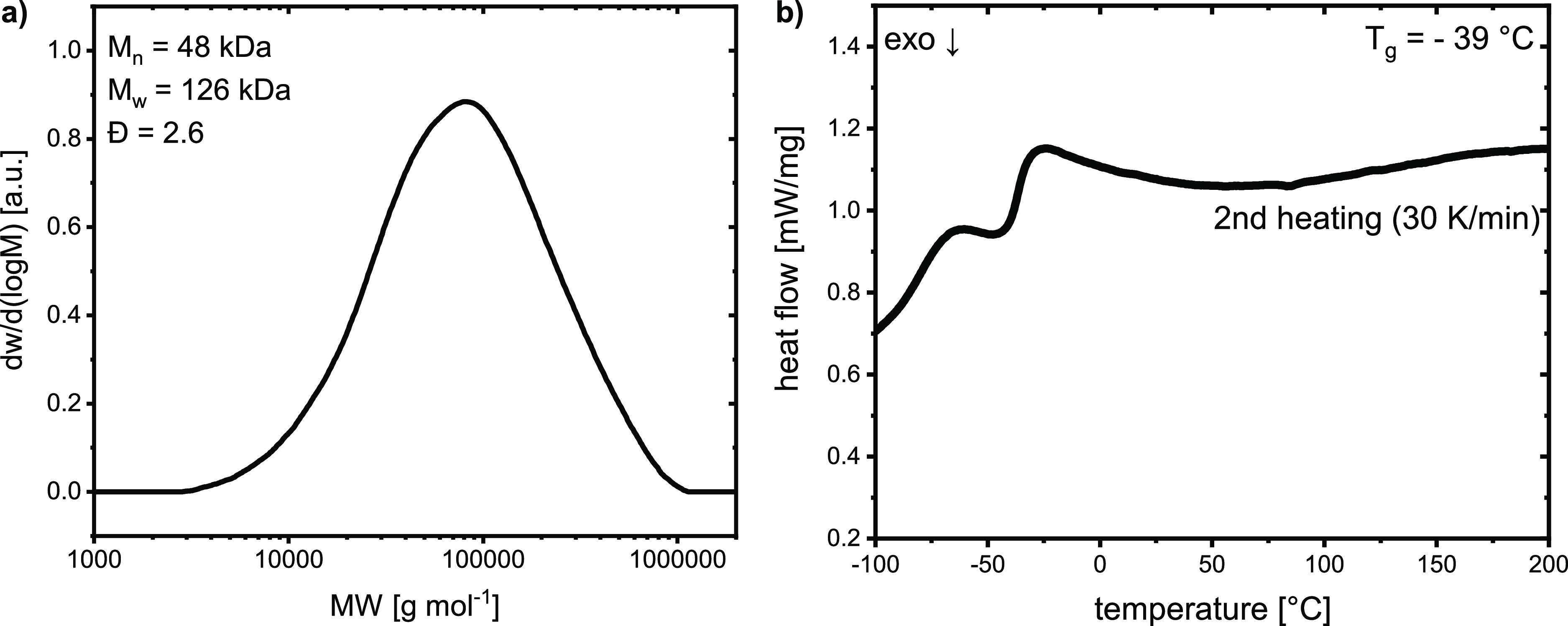
(a) SEC and (b) DSC traces of polyester **H1**.

The polycondensation of readily available biobased hydrogenated DA (C_36_) monomers with EG led to hydrophobic, amorphous polyesters with high molecular weight using a protocol previously used to produce high-molecular-weight semicrystalline polyesters from linear monomers (Figures S13 and S14). In a terpolymerization with comparably low-molecular-weight unsaturated diacids (with C_4_ being the smallest possible configuration) maleic acid (MA, C_4_) or hexenedioic acid (HA, C_6_), amorphous, unsaturated polyesters were synthesized. The melt polycondensations catalyzed with DBTO were stabilized with BHT and conducted at lower temperature than for saturated polyesters (140–160 °C, Figure 1, depicted for HA-containing polyesters) to minimize cross-linking of the double bonds. Still, reasonably high molar masses could be achieved (*M*_n_ = 48 kg/mol and *M*_w_ = 126 kg/mol, Figure 2a). An exemplary ^1^H NMR spectrum is shown in Figure S9. The saturated and unsaturated diacids were utilized in various ratios, all resulting in fully amorphous polyesters with *T*_g_ values between −45 and −40 °C (Figure 2, right, Figure S11, and Table S1). The undesired reaction of the double bonds at the elevated temperatures of polycondensation occurred only in a negligible amount for HA, as concluded from ^1^H NMR analysis of the polymers and indicated by a narrow PDI (Table S1). The double bond of MA, however, exhibited a higher reactivity, leading to some cross-linking visible in the SEC chromatograms (Figure S10). The polyester synthesized from 1 equiv of DA, 1 equiv HA, and 2 equiv EG was used as a model system for further vulcanization and is hereinafter termed polyester **H1**. The SEC trace of an upscaled polymerization with MA (polyester **M1**) is shown in Figure S10 and DSC traces are shown in Figure S11.

**Figure 3:**
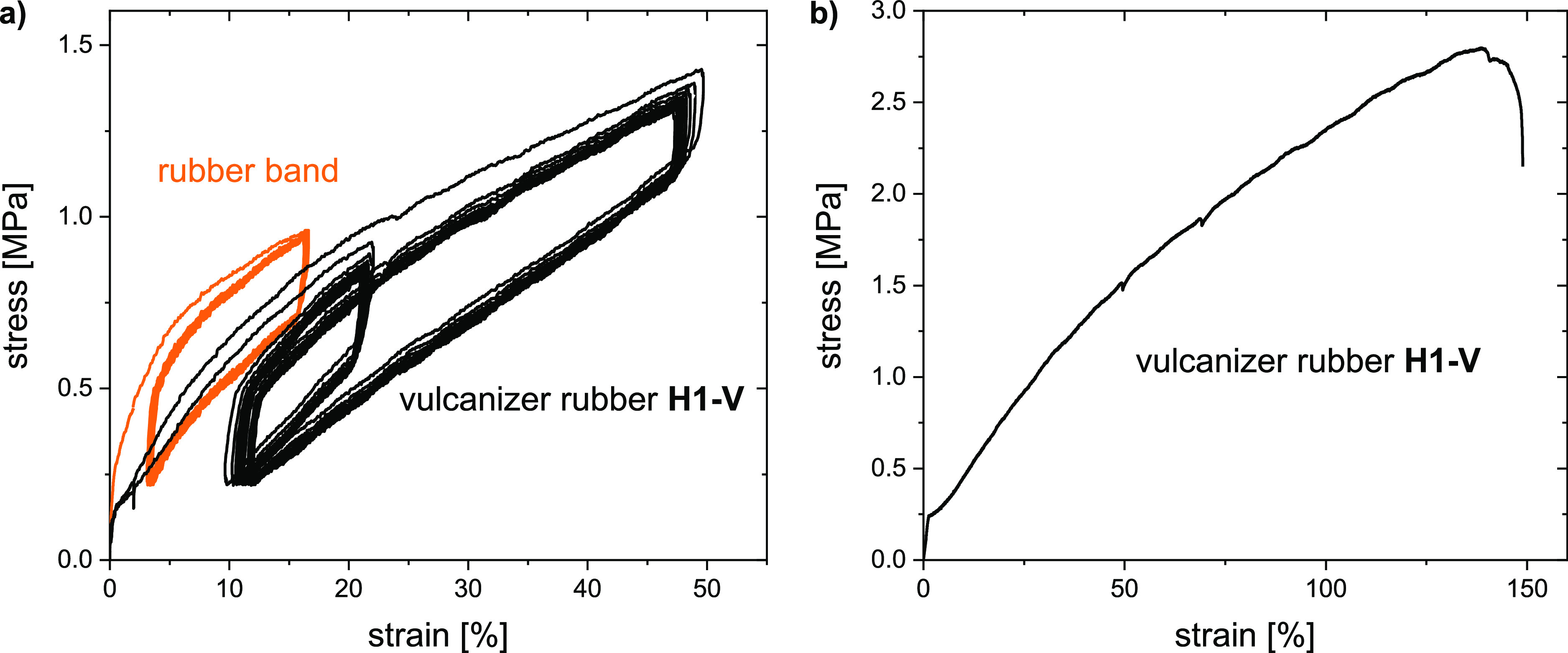
(a) Cyclic hysteresis tests of vulcanized rubber **H1-V** compared to a conventional rubber band and (b) stress–strain curves of vulcanized rubber **H1-V**.

To this end, polyester **H1**, which was a highly viscous, nonsticky substance at room temperature, was mixed with elemental sulfur, zinc oxide, and TBBS as vulcanization accelerators according to a “conventional vulcanization” protocol.^32,33^ Stearic acid, typically employed in this protocol to solubilize ZnO particles in the hydrophobic matrix, was not used here due to the assumption that the already present carboxylate and hydroxyl chain ends as well as polar ester groups would be sufficient in that regard. Indeed, manual mixing of the components at 100 °C yielded a homogeneous, white material. Vulcanizing for 1 h at 180 °C led to an elastic rubber (Materials section, Figure S1). The additional polyesters were vulcanized according to the same protocol, and their vulcanization time depended on the identity and relative amount of unsaturated comonomer used, with MA displaying the highest reactivity (Figure S2). The resulting polyester-based vulcanized rubber **H1-V** was intractable and insoluble, exhibiting a gel content of ≥90% (Table S2). Tensile testing of **H1-V** showed elastic behavior, comparable to the properties of a commercial rubber band, and an elongation at break of *ca.* 150% (Figures 3 and S12).

Depolymerization as a means of chemical recycling was performed *via* methanolysis without additional catalysts, as previously reported for polyethylene-like polyesters^29,34^ or PET.^35^ After 6 days at 150 °C, the cross-linked rubber underwent complete depolymerization into its constituent monomers. The resulting brownish solution (Figure S4, left) was filtered to remove solids such as vulcanizing agents. Subsequent separation by distillation yielded the individual monomers. The main component of the polymer, dimer dimethyl ester, was recovered *via* short-path distillation at high temperatures and high vacuum in 97% yield and a purity >99% (concluded from ^1^H NMR, Figures S5 and S6). Additionally, an EG-rich fraction was recovered (Figure S7). Notably, the unsaturated monomers (subject to the cross-linking process) and their reaction products were effectively separated, particularly from the dimer acid fraction. While the recycling conditions studied here are certainly not optimum for larger-scale operations due to the long reaction time, they can be easily improved by the use of a catalyst.^34^ Methanolysis of PET, for example, is usually performed with KOH as the catalyst at 180–280 °C^36^ and is still the topic of numerous ongoing studies.^37,38^ Note that both the methanolysis of PET^39^ and dimer acid purification *via* distillation^40^ are established procedures on an industrial scale.

As the saturated monomers do not undergo any changes during vulcanization, the recovered monomer is suitable for reutilization by polycondensation. The introduction of fresh unsaturated monomers would allow for the synthesis of a new virgin polyester. Isolation of the saturated dicarboxylate monomer, which accounts for the largest part of the material, is facilitated by its relatively high molecular weight and consequently boiling point compared to the other components. Relatively low-molecular-weight unsaturated monomers were judiciously chosen, as these are not recycled when cross-linked, to enable a high C atom recycling rate.

By recycling only the dimer acid component of vulcanized rubber **H1-V**, a theoretical recycling rate of 78% C atoms is attainable. Here, a recycling rate of 76% from monomer to monomer was demonstrated. By recovering and purifying the EG monomers as well, the theoretical recycling rate increases to 87% and, with the same monomer ratio, to 91% for MA-based materials, respectively. Lower amounts of unsaturated monomers correspond to even higher theoretical recycling rates. To this end, in preliminary experiments even with a higher molar ratio of DA to HA of 4.7, (weight ratio *ca.* 18) vulcanizable materials were obtained, corresponding to a theoretical recycling rate of up to 97% of the carbon atoms (Table S1). As in our concept, the unsaturated monomers are not reused, it is important to note that especially MA is a platform chemical,^41^ thus affordable, and HA can be produced biobased as well.^42^ Also other available unsaturated monomers such as itaconic acid or muconic acid could be used as alternative cross-linkers.

### Biodegradation and Hydrolytic Stability

The in-chain ester groups as predetermined breaking points not only facilitate chemical recycling of the polyester rubbers; in principle, they can also be hydrolyzed by abiotic or biotic means. On the other hand, such processes are expected to be impeded by the hydrophobic nature of the material. Indeed, exposing the uncured polyester **H1** to neutral aqueous buffer even at elevated temperature (phosphate buffer, pH 7.2, 75 °C, 24 h) did not result in any significant change of molecular weight (Table S3). The abiotic hydrolysis of the uncured polyester is not expected to be significantly faster than that of the vulcanized material.

**Figure 4:**
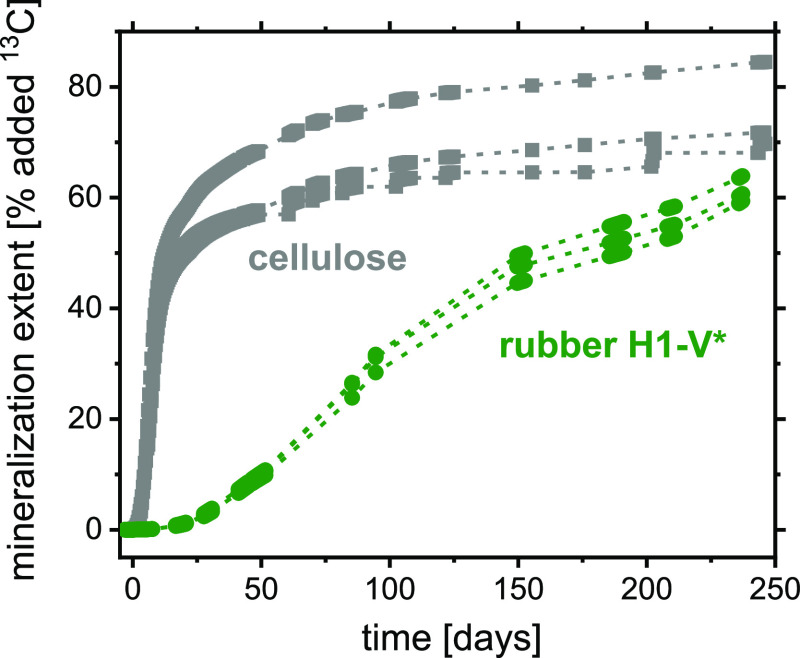
Soil biodegradation extents at 25 °C of ^13^C-labeled vulcanized rubber H1-V* compared to those of cellulose (calculated from rates shown in Figure S8). Labeling was achieved by employing ^13^C_2_–EG in the polyester synthesis. Incubations were performed in the same bulk agricultural soil and in triplicate for each material. Measured data for individual replicate incubations are shown as points, with dashed lines as linear interpolation between measurements, to guide the eye. The data on biodegradation of cellulose as a reference biodegradable polymer in the same soil has been published previously.^31^

One likely receiving environment of rubber-derived micro- or nanoplastics is soil. The soil biodegradability of the polyester-based vulcanized rubber was elucidated *via* stable isotope-selective CO_2_ tracking, a method previously established for quantitatively and unambiguously following mineralization of polymers.^43,44^ A small-scale batch of polyester H1* was synthesized employing ^13^C_2_–EG and subsequently vulcanized and manually cut into small particles (approximately 1 mm^3^). Monitoring of biodegradation in agricultural soil at 25 °C showed substantial and continual conversion of the EG monomer to ^13^CO_2_, with the mineralization extent reaching around 60% of the added EG–^13^C after 240 days (Figure 4). As not all monomers were ^13^C labeled and investigated, these experiments were not suited to classify the material as biodegradable according to, *e.g.*, ISO norms.^45,46^ Nonetheless, the mineralization of the EG units indicates an environmental hydrolysis of the polyester to its monomers, which is widely accepted as the rate-limiting step of polymer biodegradation in soil,^47,48^ followed by microbial utilization of EG-containing oligomers or free EG. Neither the cross-linked nature of the material nor the vulcanization additives prevented the substantial degradation of this rubber under mild conditions. While also conventional natural and synthetic rubbers can be degraded by certain microorganisms,^5^ most studies have demonstrated this using isolated strains under idealized conditions,^49,50^ while the degradation behavior of rubbers in nonspecific, natural environments is less well investigated. The limited evidence available indicates that tire wear particles, mainly consisting of conventional synthetic rubbers, are nearly inert in aquatic environments, showing close to zero CO_2_ evolution over 80 days.^51^ Further experiments are needed to investigate the biodegradation of the rubbers presented here, especially to investigate the microbial utilization of the different components, such as dimer acid and sulfur-containing monomer units. Furthermore, experiments comparing the biodegradability of polyester-based rubbers and conventional rubbers in the natural environment would be instructive. Note that remediation of micro- or nanoplastics will be facilitated by the particles’ high surface area,^52,53^ which facilitates surface degradation processes (as opposed to bulk degradation mechanisms) like enzymatic degradation in particular.^54^

## Conclusions

The polyester-based rubbers reported here are chemically recyclable and biodegradable while maintaining the characteristics of covalently cross-linked elastomers formed *via* conventional vulcanization procedures. Upscaling of the materials and processes shown herein is promising as the synthetic procedures are well-established, and readily available monomers were used exclusively. In particular, the theoretical chemical recycling rates exceeding 90% constitute a substantial advancement from the status quo of chemical recycling of conventional rubbers. As the monomers used herein are biobased or can be synthesized from biobased resources, these materials would also contribute to a shift of the chemical industry to renewable feedstocks.^24,25^

The recyclability and biodegradability of rubbers are particularly crucial for tires, as one billion tires exceed their operational lifetime every year generating massive amounts of waste, and tire wear particles are inevitably introduced into the environment in a million ton range every year,^52,55^ making them the major source of microplastics in some environments.^55^ Considering the potential impact of both the particles and leaching additives on human health and wildlife^56,57^ and with the anticipated increase in tire runoff due to increased volume of traffic, the development of environmentally benign rubber materials is desirable. Notably, chemical recycling of tires enables the recovery of not only the rubber but also valuable fillers and reinforcement materials such as carbon black and steel. Tires are highly engineered products optimized over decades with key metrics like wet traction, rolling resistance and abrasion resistance.^58^ As they are subject to safety requirements, new materials would need extensive testing. Anticipated next steps comprise the upscaling of production for applying more advanced processing methods, such as compression molding. Detailed studies of the viscosity behavior of the uncured rubber and curing kinetics as well as long-term stability experiments to determine the biodegradability of a final compound and the stability under stress are required to establish the processability of this material with existing equipment and applications, such as tires.

## Supporting Information Available

Supplementary methods and data, including NMR, SEC, DSC, and tensile testing data of different amorphous polyesters and vulcanizates and additional data concerning vulcanization times and hydrolytic stability (PDF)

## Notes

A patent has been filed by S.T.S. and S.M. on the findings reported here.
