# Supplementary material for: Chemically Recyclable and Biodegradable Vulcanized Rubber
Source: ACS Sustain Chem Eng. 2024 Apr 11;12(16):6281–8. doi: 10.1021/acssuschemeng.3c08435 (PMC11041115; doi:10.1021/acssuschemeng.3c08435)
Supplement: Supplementary file 1 — sc3c08435_si_001.pdf [file sc3c08435_si_001.pdf]

Chemically Recyclable and Biodegradable Vulcanized Rubber

Supporting Information

Simon T. Schwab, Taylor F. Nelson, Stefan Mecking*

Chair of Chemical Materials Science, Department of Chemistry, University of Konstanz

Number of pages: 14

Number of figures: 14

Number of tables: 3

Table of contents

Polymerization Experiments 1
Vulcanization Experiments 2
Gel Content Determination 4
Recycling Experiments 5
Hydrolytic Stability 8
Biodegradation rates 8
Further Analytical Data of Hexenedioic Acid-Based Polyester **H1** 9
Further Analytical Data of Maleic Acid-Based Polyester **M1** 10
Further Analytics of Dimer Acid Ethylene Glycol Copolymer 12

Polymerization Experiments

Table S 1: Average molar masses and monomer compositions of unsaturated, amorphous polyesters.

Sample	Unsat. diacid	M_n (NMR) [kg/mol]	Ratio sat. : unsat. diacid	Unreacted unsat. monomer [%]	M_n (SEC) [kg/mol]	M_w (SEC) [kg/mol]	Theoretical recycling rate [%]
**H1**	Hexene dioic	40	1.2	100	48	126	89
**H2**	Hexene dioic	15	1.2	100	19	36	89
**H3**	Hexene dioic	14	2.3	100	16	30	94
**H4**	Hexene dioic	24	4.7	100	16	30	97
**M1**	Maleic	8.1	1.4	95	25	128	93
**M2**	Maleic	7.2	1.7	75	20	53	94
**M3**	Maleic	14	4.4	90	26	72	98

Vulcanization Experiments

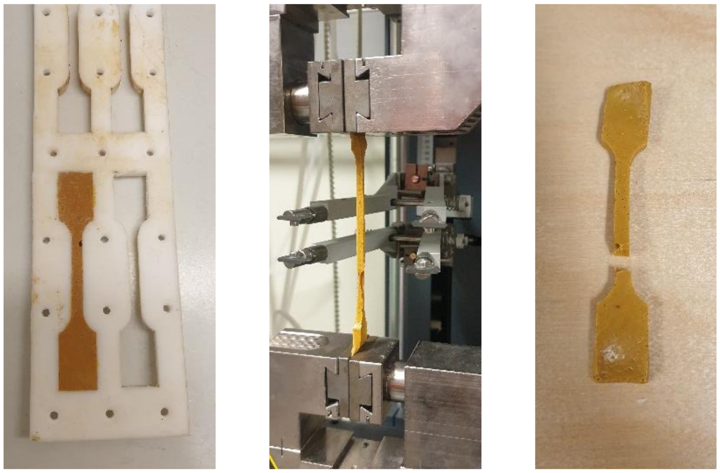

Figure S 1: Vulcanized rubber tensile testing specimen **H1-V** in PTFE mold (left), cyclic hysteresis experiment of tensile testing specimen (center), and tensile testing specimen after tensile testing experiment (right).

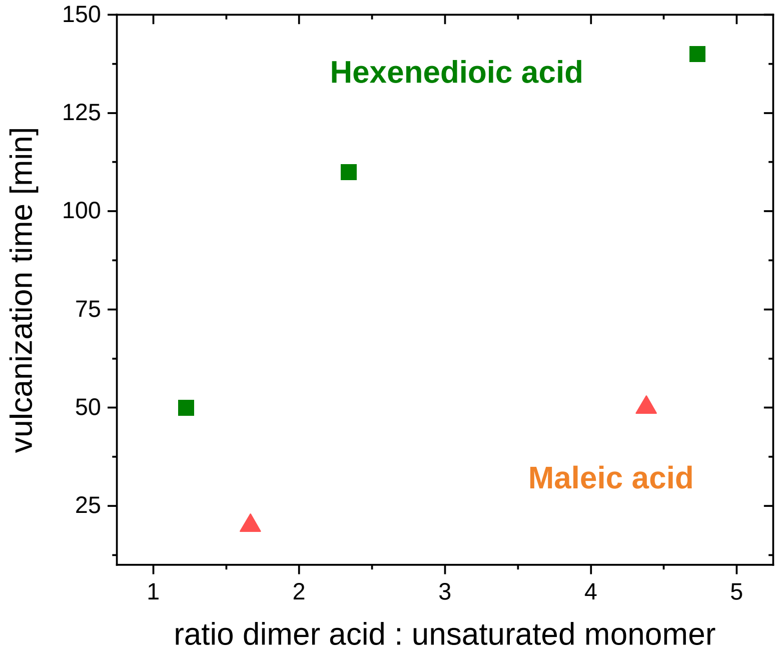

Figure S 2: Vulcanization times of polyesters consisting of different unsaturated acids and exhibiting different double bond densities employing a 'conventional vulcanization' protocol. Vulcanization times were qualitatively determined by visual inspection of the samples, and defined as the time required for a given sample to transition from a viscous liquid to an elastic solid.

Gel Content Determination

Table S 2: Sol contents of three replicate samples of one vulcanized rubber, determined by immersing in chloroform at 37 °C for 6 hours.

Sample	Sample mass [mg]	Remaining mass [mg]	Sol content [%]
Rubber **H1-V** a	44.4	3.6	8
Rubber **H1-V** b	36.6	3.7	10
Rubber **H1-V** c	47.7	5.5	12

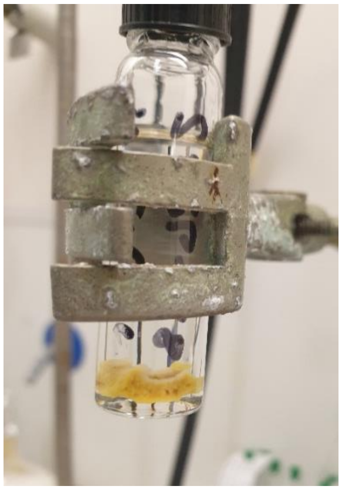

Figure S 3: A piece of **H1-V** in 100 °C toluene.

Recycling Experiments

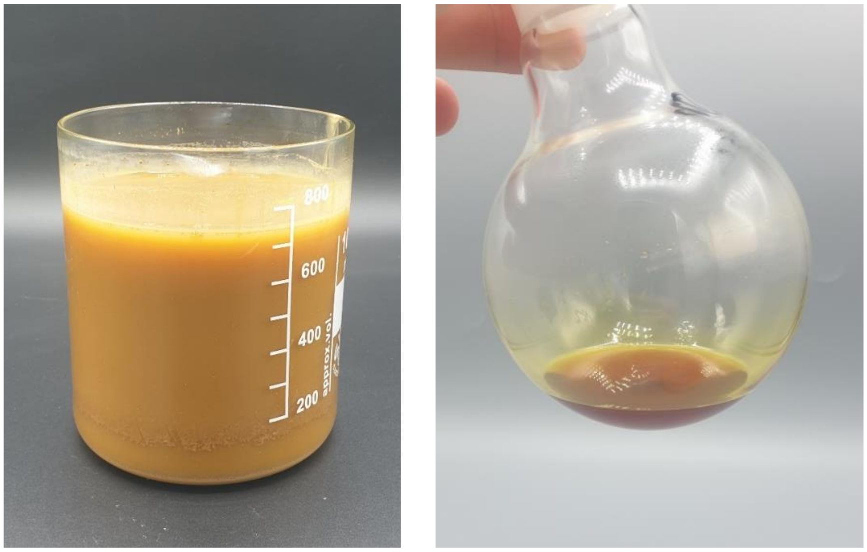

Figure S 4: Monomer mixture as obtained from the methanolysis (left) and distilled and recycled dimer diester monomer (right).

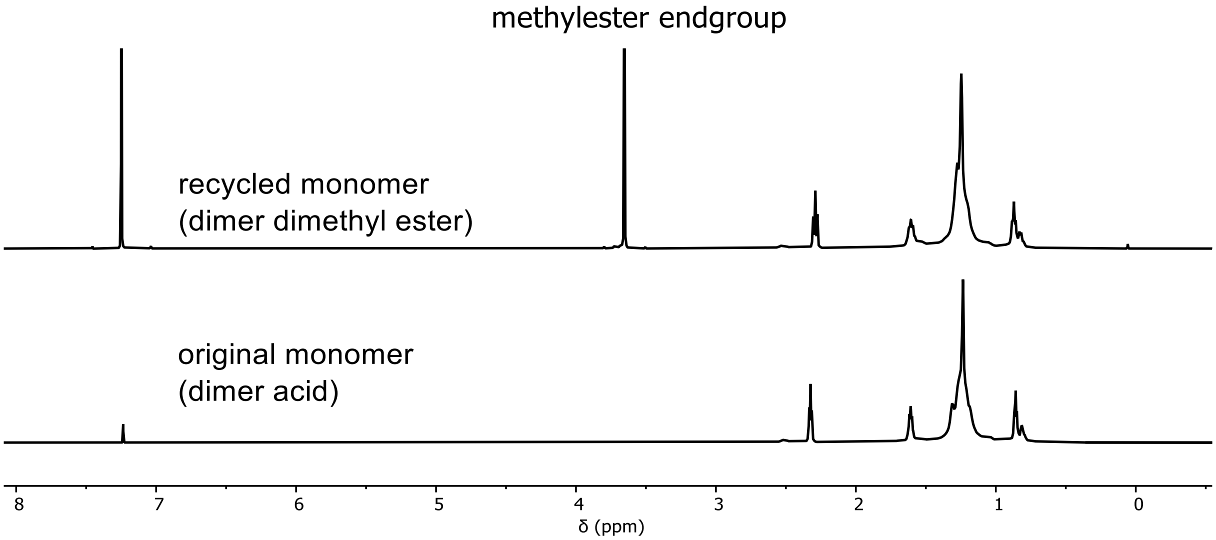

Figure S 5: ^{1}H NMR (400 MHz, $CDCl_3$, 300 K) spectra of virgin dimer acid monomer (bottom) and the recycled dimer dimethyl ester monomer (top).

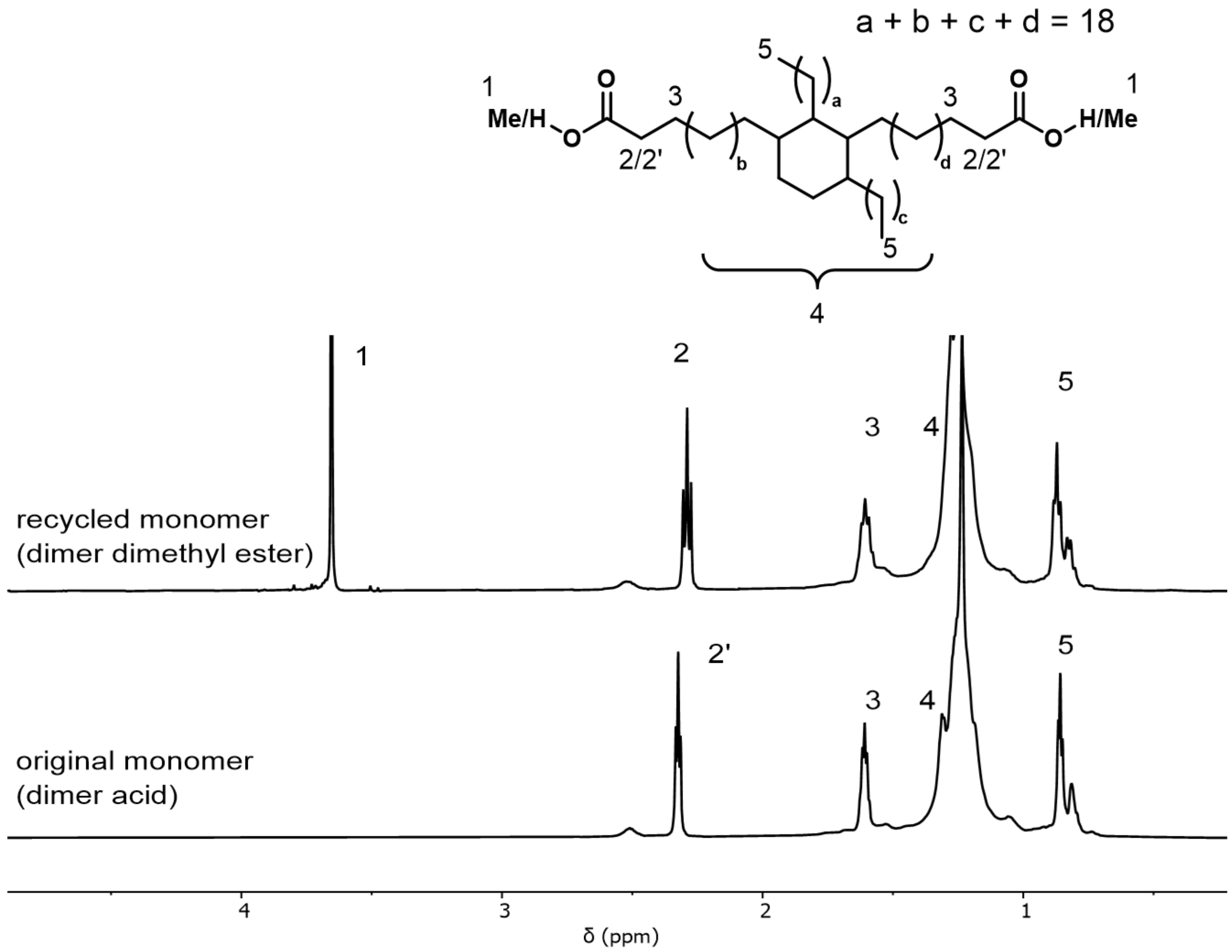

Figure S 6: Details of ^{1}H NMR (400 MHz, $CDCl_3$, 300 K) spectra of virgin dimer acid monomer (bottom) and the recycled dimer dimethyl ester monomer (top).

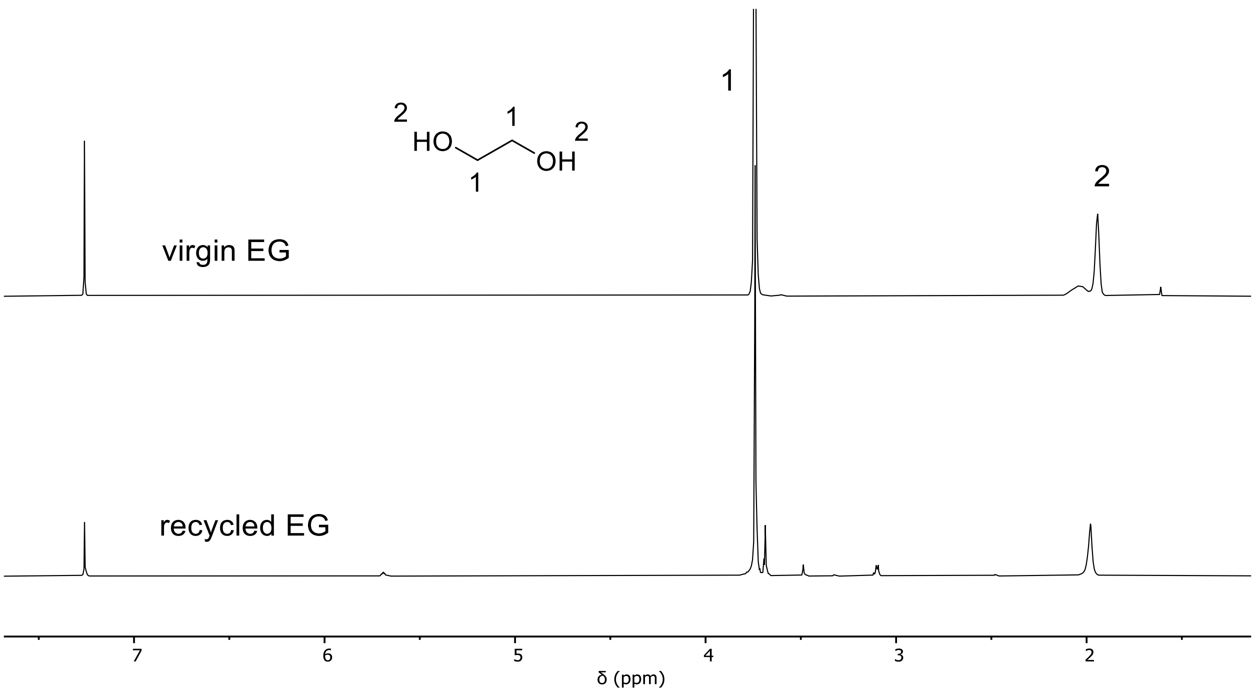

Figure S 7: ^{1}H NMR (400 MHz, $CDCl_3$, 300 K) spectra of virgin ethylene glycol (top) and retrieved EG-rich fraction upon recycling (bottom).

Hydrolytic Stability

Table S3: Molar masses of the non-cured amorphous, unsaturated polyesters before and after testing for hydrolytic stability in phosphate buffer, pH 7.2.

Sample	M_n [kg/mol]	M_w [kg/mol]	M_w/M_n
Virgin	33	132	3.8
Buffer at 25 °C	33	150	4.6
Buffer at 75 °C	32	121	3.8

Biodegradation rates

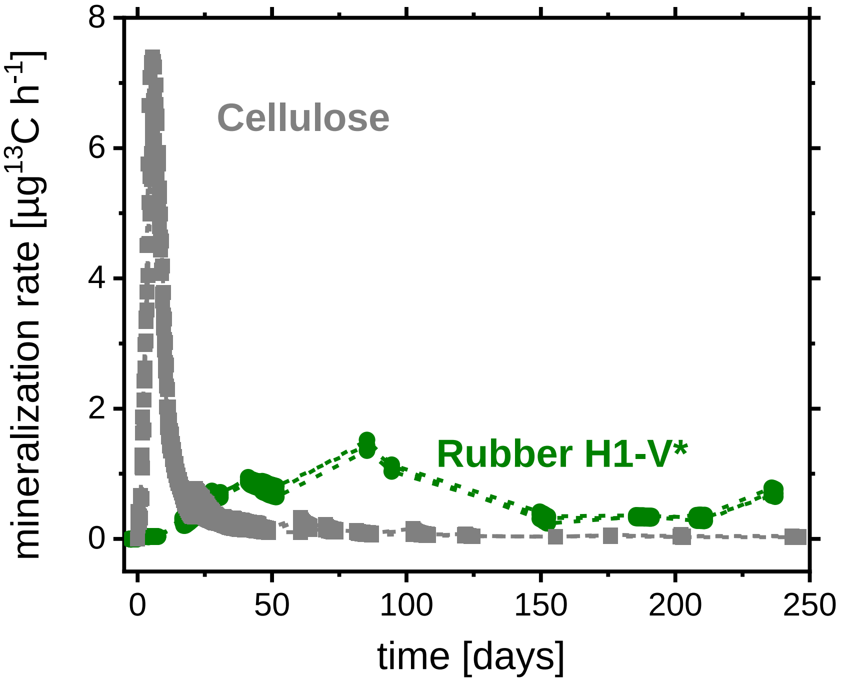

Figure S 8: Mineralization rates at 25 °C of ^{13}C labelled vulcanized rubber **H1-V*** compared to those of cellulose.

Further Analytical Data of Hexenedioic Acid-Based Polyester **H1**

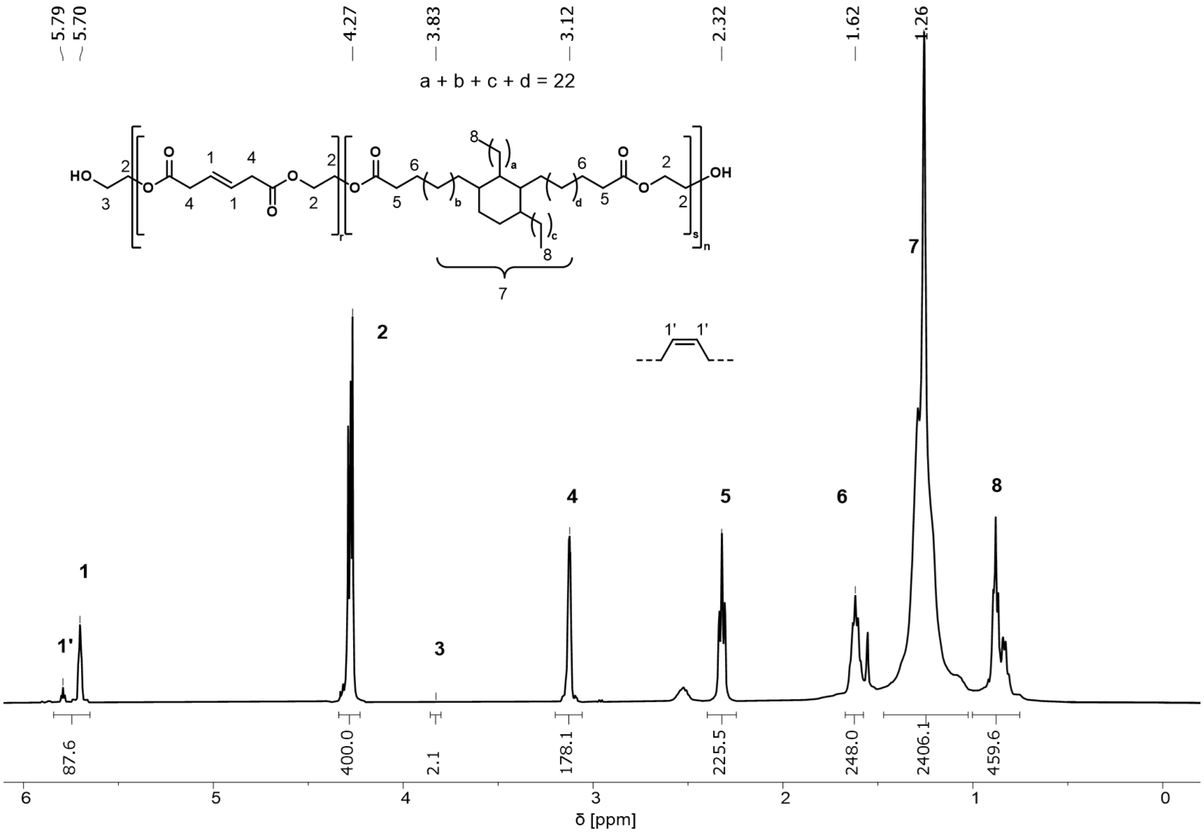

Figure S 9: ^{1}H NMR (400 MHz, $CDCl_3$, 300 K) spectrum of the polyester **H1**.

Further Analytical Data of Maleic Acid-Based Polyester **M1**

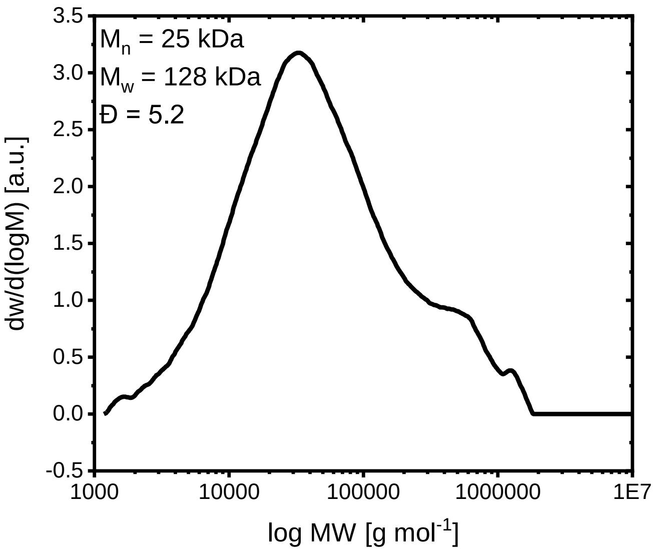

Figure S 10: SEC trace of the polyester **M1**.

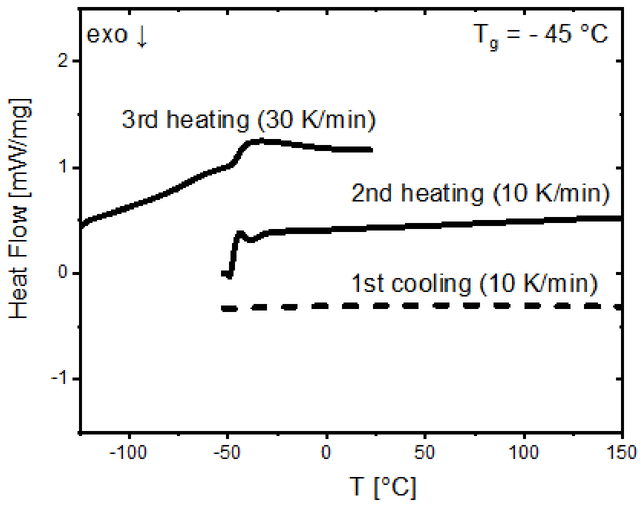

Figure S 11: DSC analysis of the polyester **M1**. Second heating and first cooling traces were acquired at a rate of 10 K min^{-1} to determine the melting point, and third heating was performed at 30 K min^{-1} to determine the glass transition temperature.

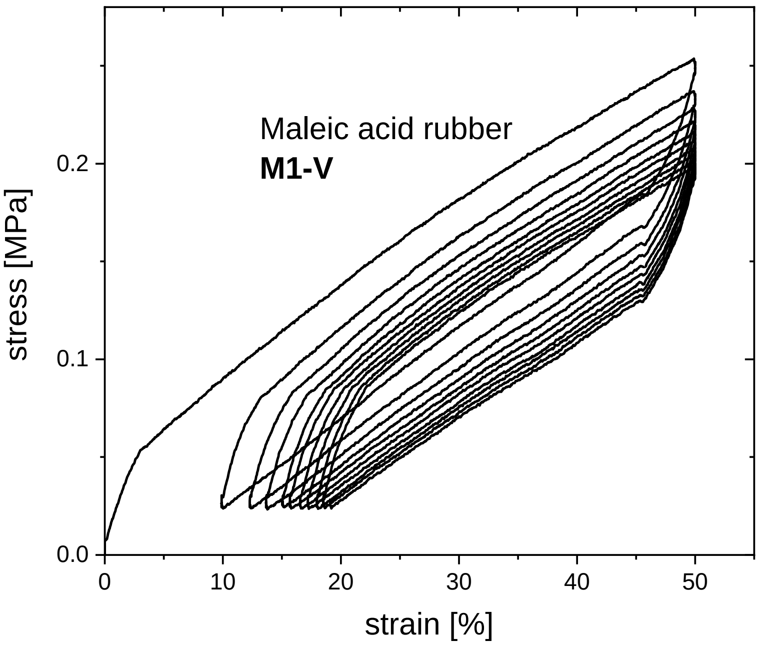

Figure S 12: Cyclic hysteresis tests of the vulcanized rubber **M1-V**.

<u>Further Analytics of Dimer Acid Ethylene Glycol Copolymer</u>

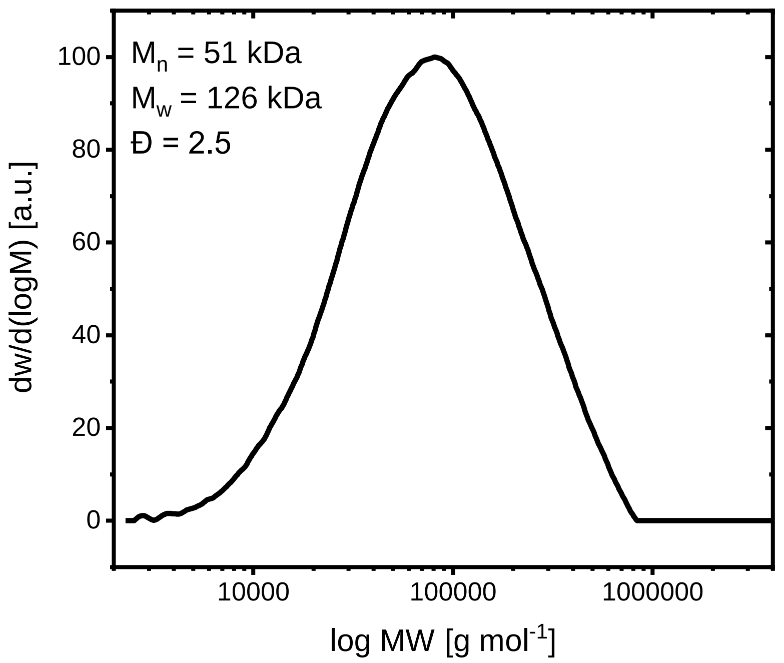

Figure S 13: SEC trace of dimer acid-ethylene glycol based polyester.

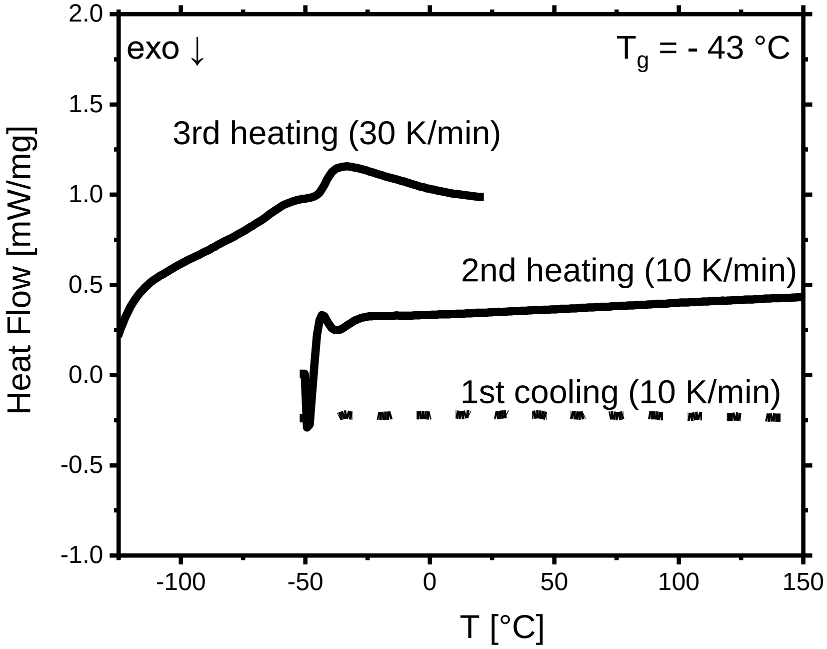

Figure S 14: DSC traces of dimer acid-ethylene glycol based polyester.
